# Continuous and intermittent theta burst stimulation to the visual cortex do not alter GABA and glutamate concentrations measured by magnetic resonance spectroscopy

**DOI:** 10.1002/brb3.2478

**Published:** 2022-01-14

**Authors:** Karlene S. Stoby, Sara A. Rafique, Georg Oeltzschner, Jennifer K. E. Steeves

**Affiliations:** ^1^ Centre for Vision Research and Department of Psychology York University Toronto ON Canada; ^2^ Russell H. Morgan Department of Radiology and Radiological Science The Johns Hopkins University School of Medicine Baltimore Maryland USA; ^3^ F. M. Kirby Research Center for Functional Brain Imaging Kennedy Krieger Institute Baltimore Maryland USA

**Keywords:** gamma‐aminobutyric acid, glutamate, magnetic resonance spectroscopy, theta burst stimulation, transcranial magnetic stimulation, visual cortex

## Abstract

**Background:**

Theta burst stimulation (TBS), a form of repetitive transcranial magnetic stimulation (rTMS), uses repeated high‐frequency bursts to non‐invasively modulate neural processes in the brain. An intermittent TBS (iTBS) protocol is generally considered “excitatory,” while continuous TBS (cTBS) is considered “inhibitory.” However, the majority of work that has led to these effects being associated with the respective protocols has been done in the motor cortex, and it is well established that TMS can have variable effects across the brain.

**Objectives and method:**

We investigated the effects of iTBS and cTBS to the primary visual cortex (V1) on composite levels of gamma‐aminobutyric acid + co‐edited macromolecules (GABA+) and glutamate + glutamine (Glx) since these are key inhibitory and excitatory neurotransmitters, respectively. Participants received a single session of cTBS, iTBS, or sham TBS to V1. GABA+ and Glx were quantified in vivo at the stimulation site using spectral‐edited proton magnetic resonance spectroscopy (^1^H‐MRS) at 3T. Baseline pre‐TBS GABA+ and Glx levels were compared to immediate post‐TBS and 1 h post‐TBS levels.

**Results:**

There were no significant changes in GABA+ or Glx following either of the TBS conditions. Visual cortical excitability, measured using phosphene thresholds, remained unchanged following both cTBS and iTBS conditions. There was no relationship between excitability thresholds and GABA+ or Glx levels. However, TBS did alter the relationship between GABA+ and Glx for up to 1 h following stimulation.

**Conclusions:**

These findings demonstrate that a single session of TBS to the visual cortex can be used without significant effects on the tonic levels of these key neurotransmitters; and add to our understanding that TBS has differential effects at visual, motor, and frontal cortices.

## Graphical abstract

iTBS and cTBS protocols applied to V1 had no significant effect on local GABA and glutamate concentrations. TBS did alter the relationship between GABA and glutamate levels for at least 1 h following stimulation. Results demonstrate that TBS produces differential effects at the visual cortex compared to aftereffects that have been measured at motor and frontal cortices. These findings have key implications for clinical use, and for investigative vision research when making inferences on causality.

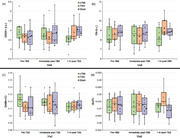

## 1 INTRODUCTION

Non‐invasive brain stimulation (NIBS) techniques such as repetitive transcranial magnetic stimulation (rTMS) are an invaluable tool to induce neural changes safely and effectively across brain networks in healthy and patient populations. TMS uses strong focused magnetic field pulses to induce lasting neural changes at the stimulation site and remote regions (Hallet, 2007; Kobayashi & Pascual‐Leone, 2003). Theta burst stimulation (TBS), a type of high‐frequency rTMS, is increasingly being used as an alternative to conventional rTMS protocols since it can be delivered more quickly while achieving similar outcomes as conventional protocols (Blumberger et al., 2018; Suppa et al., 2016). Intermittent TBS (iTBS) typically generates an “excitatory” response, due to its interval pattern, in a similar fashion to long‐term potentiation (LTP) of synaptic transmission in the brain. Continuous TBS (cTBS) typically produces an “inhibitory” response, due to its continuous pattern, mimicking a reaction like long‐term depression (LTD) (Hess et al., 1996; Huang et al., 2005). However, this dichotomy that iTBS is excitatory and cTBS is inhibitory is inadequate since it has been shown that both high‐ and low‐frequency rTMS can have mixed excitatory and inhibitory effects (Houdayer et al., 2008). Even when the rTMS effect appears specific, doubling the duration of stimulation can reverse the outcome from inhibition to excitation and vice versa (Gamboa et al., 2011, 2010; Goldsworthy et al., 2015). Similar adverse effects are reported between rTMS and TBS protocols (Blumberger et al., 2018). If TBS performs comparably to rTMS, then employing TBS over rTMS would considerably improve capacity, compliance, and cost by enabling stimulation over shorter sessions. This would have significant implications for both experimental and clinical use.

The majority of work investigating the underlying effects of rTMS protocols have studied motor and frontal cortices (for reviews, see Hoffman & Cavus, 2002; Ridding & Ziemann, 2010; Sandrini et al., 2011; Suppa et al., 2016). Accordingly, the use of rTMS to modulate disorders has focused on these cortical regions, for example, stroke (for reviews, see Dionisio et al., 2018; Smith & Stinear, 2016; Webster et al., 2006), and psychiatric disorders (for reviews, see Ferrarelli & Phillips, 2021; Guo & Wang et al., 2017), respectively. Despite extensive research on motor and frontal cortices, the effects of TMS at the visual cortex are relatively under‐investigated and, therefore, poorly understood. Although rTMS has been used frequently in exploratory vision research (e.g., Bona et al., 2014; Chiou & Ralph, 2016; Groen et al., 2021; Julian et al., 2016; Rafique et al., 2015; Solomon‐Harris et al., 2016), very few studies have investigated the underlying mechanisms and aftereffects of rTMS, particularly TBS protocols, at the visual cortex. The use of TMS to the occipital cortex, whether clinical or investigative therefore relies on the assumption of underlying neurophysiological effects determined mainly from regions outside of the occipital cortex (Cárdenas‐Morales et al., 2010; Hallet, 2007; Hoogendam et al., 2010; Thut & Pascual‐Leone, 2010). However, underlying neurophysiological effects of NIBS differ fundamentally across cortical and subcortical regions (Castrillon et al., 2020). As a result, rTMS applications are less widely used in the clinical application of visual‐related disorders compared to non‐visual‐related conditions. Clinically, TMS has great potential as a valuable therapeutic tool in several visual and ophthalmological disorders (Mahayana et al., 2017). Previously, we have successfully reduced visual hallucinations that occurred as a consequence of occipital stroke using rTMS to the visual cortex (Rafique et al., 2016). Others have used rTMS to manage visual hallucinations from Charles Bonnet syndrome (Merabet et al., 2003). TBS to the posterior parietal cortex improves visual‐spatial neglect in patients with right‐hemispheric stroke (Cazzoli et al., 2012). Both rTMS and TBS protocols to the occipital cortex have significantly improved visual acuity, stereoacuity, and contrast sensitivity measures in amblyopia (Clavagnier et al., 2013; Thompson et al., 2008; Tuna et al., 2020).

Neurotransmitters such as γ‐aminobutyric acid (GABA) and glutamate are key actors in inhibitory and excitatory neural processes corresponding to LTD‐ and LTP‐like changes (for a review, see Lüscher & Malenka, 2012) and provide insight into TMS mechanisms. Levels of neurotransmitters and other metabolites can be measured non‐invasively in vivo using magnetic resonance spectroscopy (MRS). Although MRS is not able to distinguish between vesicular and synaptic or intra‐ and extracellular pools, instead, it quantifies “bulk” tonic levels averaged over a macroscopic region of interest. MRS‐measured GABA contains co‐detected GABA‐like metabolites like homocarnosine and macromolecules with similar spectra. The composite measure is consequently referred to as GABA+. Similarly, MRS‐measured glutamate contains the co‐detected glutamine signal, and the composite signal is referred to as Glx (Ramadan et al., 2013; Schmidt‐Wilcke et al., 2018). Table 1 provides an overview of studies investigating the effects of rTMS on MRS‐measured GABA and glutamate in healthy participants. Although there is a lack of data investigating changes in metabolites following rTMS, Table 1 highlights variability in findings across studies owing to differences in stimulation parameters and the cortical region being stimulated. We previously investigated how a single‐session of low‐frequency rTMS and multiple sessions within a day (termed accelerated/within‐session) influence GABA and glutamate at the visual cortex. We found that accelerated sessions significantly reduced GABA+ at the stimulation site for up to 24 h, whereas a single rTMS session had no effect, and Glx remained unchanged with both protocols (Rafique & Steeves, 2020). If TBS offers similar effects to rTMS but with shorter stimulation, then based on our previous work, we would expect a single session of cTBS to have no effect on GABA and glutamate at the visual cortex. However, Allen et al. (2014) have found that a session of cTBS significantly increased GABA in V1, while Glx was not measured and iTBS was not investigated. To progress our previous work and further the advancement of rTMS protocols in experimental and clinical vision applications, we investigated the effects of cTBS and iTBS protocols to V1 on GABA and glutamate levels, which remains unknown. For TBS to be valuable in investigative research and be implemented successfully in clinical applications through neuroplasticity changes, its effects need to be studied in various brain regions in both healthy and patient populations. Examining the underlying neurophysiological mechanisms associated with TBS will further our understanding of the various protocols and their potential therapeutic benefit. In addition, it will help establish safety profiles for various protocols and populations.

**TABLE 1:** Overview of MRS‐measured GABAergic and/or glutamatergic changes following TBS or conventional rTMS in healthy participants

Authors	Stimulation protocol	Stimulation site (SS); MRS voxel position (VP)	Sample population	Exclusion/inclusion criteria	Effect on GABA and/or Glx
Bridges et al., 2018	1 Hz rTMS (20 min [120 trains of 10 pulses, 1 s ISI, total 1200 pulses]) + Sham (placebo coil) 100% RMT	SS & VP: Left DLPFC	*N* = 11 (M_age_ = 29.6 ± 6.2 years, 10 M/1F, 9 right‐handed/2 left‐handed)	No history of cardiovascular or neurological disorders, no head trauma, no medications lowering seizure threshold, no sleep disorders No alcohol/drug abuse No alcohol 24 h before rTMS Minimum 6 h sleep before rTMS	No change in Glx GABA not measured
Iwabuchi et al., 2017	Accelerated iTBS (three iTBS sessions with 5 min rest intervals [total 1800 pulses]) + Sham (placebo coil) 80% RMT	SS: Left DLPFC VP: Left DLPFC, ACC	*N* = 27 (M_age_ = 25.1 ± 7.1 years)	No history of neurological or psychiatric disorders, no head trauma, no current medications No substance dependence	GABA/Glx ratio significantly decreased in left DLPFC and ACC following iTBS compared to sham No change in GABA/Cr or Glx /Cr
Michael et al., 2003	Multi‐day 20 Hz rTMS (five daily rTMS sessions, each 20 min [20 × 2 s trains, 58 s ISI, 800 pulses daily for a total 4000 pulses]) + Sham (coil oriented at 90° tilt) 80% AMT	SS: Left DLPFC VP: Left and right DLPFC, left ACC	*N* = 12 (rTMS M_age_ = 47.0 ± 14.0 years, 5 M/2F; sham M_age_ = 45.2 ± 12.0 years, 2 M/3F; all right‐handed)	No neurological or psychiatric disorders No history of substance abuse	Glx significantly decreased in left DLPFC following 1‐day rTMS compared to baseline; Glx significantly increased in left DLPFC following 5‐day compared to 1‐day rTMS but not baseline Glx significantly increased in right DLPFC and left ACC following 5‐day compared to 1‐day rTMS but not baseline GABA not measured
Vidal‐Piñeiro et al., 2015	iTBS + cTBS + Sham (placebo coil) ?% MT	SS: Left IPL VP: Left IPL, PCC	*N* = 31 (M_age_ = 23.5 ± 2.0 years, right‐handed) IPL: iTBS *n* = 10, cTBS *n* = 10, sham *n* = 10; PCC: iTBS *n* = 10, cTBS *n* = 10, sham *n* = 11	No neurological or psychiatric disorders Gender, age, and education matched	GABA significantly increased in PCC following iTBS compared to cTBS and sham No change at left IPL No change in Glx
Gröhn et al., 2019	1 Hz rTMS (20 min [120 trains of 10 pulses, 1 s ISI, total 1200 pulses]) + 5 Hz rTMS (22 min [24 trains of 25 pulses, 45 s ISI, total 600 pulses]) 90% RMT	SS: Left M1 VP: Left and right M1	*N* = 7 (M_age_ = 27.0 ± 7.0 years, all male, right‐handed) 1 Hz rTMS *n* = 7, 5 Hz rTMS *n* = 1	No history of cardiovascular, neurological, or psychiatric disorders, no head trauma, no current medications, no sleep apnoea No substance abuse	GABA significantly increased in left M1 and decreased in right M1 following 1 Hz rTMS compared to baseline Opposite change in GABA seen following 5 Hz rTMS No change in glutamate or glutamine
Stagg et al., 2009	cTBS + Sham (vertex stimulation) 80% AMT	SS & VP: Left M1	*N* = 15 (M_age _= 27.5 years, all male, right‐handed) cTBS *n* = 7, sham *n* = 8		GABA significantly increased in left M1 following cTBS compared to sham No change in Glx
Allen et al., 2014	cTBS + Sham (coil orientated horizontally with spacer) 80% RMT	SS & VP: V1	*N* = 18 (M_age_ = 26.3 ± 5.0 years, 11 M/7F)	Neurologically healthy	GABA significantly increased in V1 following cTBS compared to sham Glx not measured
Rafique & Steeves, 2020	Single‐session 1 Hz rTMS (20 min [120 trains of 10 pulses, 1 s ISI, total 1200 pulses]) + Accelerated 1 Hz rTMS (five 20 min rTMS sessions with 15 min rest intervals [total 6000 pulses]) 100% PT	SS & VP: V1	*N* = 16 (M_age_ = 25.2 ± 1.2 years, 10 M/6F, right‐handed, normal or corrected‐to‐normal vision) Single‐session *n* = 8, accelerated sessions *n* = 8	No underlying medical conditions, no history of neurological or psychological disorders or frequent/chronic migraines, no current medications No alcohol/substance dependence history, non‐smokers No alcohol 48 h before each visit	GABA+ significantly decreased in V1 following accelerated rTMS compared to baseline, and effects lasted up to 24 h; no change in Glx No change in GABA+ or Glx following single‐session rTMS

*Note*. cTBS consists of bursts containing three pulses at 50 Hz (20 ms between each stimulus), repeated at 5 Hz intervals (i.e., 200 ms ISI), applied continuously/uninterrupted for 40 s, providing a total of 600 pulses; and iTBS consists of the same bursts containing three pulses at 50 Hz, repeated at 5 Hz intervals but applied in 2 s trains repeated every 10 s for a total of 190 s, also providing a total of 600 pulses (Huang et al., 2005). MRS = magnetic resonance spectroscopy; TBS = theta burst stimulation; cTBS = continuous TBS; iTBS = intermittent TBS; rTMS = repetitive transcranial magnetic stimulation; AMT = active motor threshold; RMT = resting motor threshold; PT = phosphene threshold; ISI = inter‐stimulation interval; *N* = total sample size; *n* = subset sample size; M_age_ = mean age; M = male, F = female; GABA = γ‐aminobutyric acid; GABA+ = γ‐aminobutyric acid + co‐edited macromolecules; Glx = glutamate + glutamine; Cr = creatine; DLPFC = dorsolateral prefrontal cortex; ACC = anterior cingulate cortex; IPL = inferior parietal lobe; PCC = posterior cingulate cortex; M1 = primary motor cortex; V1 = primary visual cortex.

## 2 METHODS

### 2.1 Participants

We recruited 39 healthy right‐handed participants aged 18−35 years. Participants had no known contraindications to TMS and magnetic resonance imaging (MRI), no underlying medical conditions, and no history of neurological or psychological disorders (Kim et al., 2019; Levinson et al., 2010; Schür et al., 2016). We employed further strict exclusion criteria to control for confounding factors and to minimize potential extraneous interactions associated with metabolite receptors and/or TMS mechanisms. Participants were not taking any medications at the time of participation (Stell et al., 2003) including hormonal contraceptives (Kaore et al., 2012; Smith et al., 2002), had no history of frequent or chronic migraines (Bohotin et al., 2002; Russo et al., 2005), no history of alcohol/substance dependence (Brust, 2004; Ke et al., 2004; Lobo & Harris, 2008; Malcolm, 2003), and were non‐smokers (Epperson et al., 2005). Additionally, participants were asked to attempt a good night's sleep (Clow et al., 2014), and not to consume alcohol 48 h (Lobo & Harris, 2008) before each visit. Participants were assigned to one of three experimental groups (cTBS, iTBS, sham) in a pseudo‐random fashion and were naive to the stimulation condition. Ten participants were discarded due to high MRS data fit errors, motion artifacts, or failure to meet exclusion criteria following the initial visit/screening. The remaining participants (mean_age_ ± SD = 22.17 ± 3.52 years; *N* = 29; 12 males/17 females) had normal or corrected‐to‐normal vision. All participants gave informed consent, and the protocol was approved by the Office of Research Ethics at York University in accordance with the Declaration of Helsinki. Participants received monetary compensation.

### 2.2 Vision and cognitive assessments

All participants were required to complete and pass three basic visual assessments to ensure eligibility for normal or corrected‐to‐normal vision (>0.04 logMAR; stereoacuity ≥ 50″, normal color vision). Monocular and binocular visual acuities were measured using the standardized ETDRS logMAR vision chart (Precision Vision, La Salle, IL), stereo acuity was measured using the Titmus circles test (Stereo Optical Company Inc., Chicago, IL), and color vision were assessed using the Ishihara test (Kanehara Trading Inc., Tokyo, Japan).

All participants completed and passed the Montreal Cognitive Assessment (MoCA, v7.1−7.3; Nasreddine et al., 2005) screening for detecting mild to severe cognitive impairment. The MoCA evaluates attention, concentration, working memory, short‐term memory, delayed recall, language, visuospatial, orientation, and executive function. Participants completed different versions at each visit.

### 2.3 Magnetic resonance imaging

Anatomical and MRS data were acquired with a 3 Tesla Siemens Magnetom® Prisma magnetic resonance scanner with a 32‐channel high‐resolution array head coil (Siemens, Erlangen, Germany). Anatomical high‐resolution T_1_‐weighted images were acquired first followed by MRS. Participants were instructed to remain still, keep their eyes closed throughout, and refrain from falling asleep. Imaging was performed with the room lights turned off.

#### 2.3.1 Anatomical T_1_‐weighted

The T_1_‐weighted magnetisation‐prepared rapid gradient echo (MPRAGE) imaging sequence was acquired with the following parameters: number of slices = 192, in‐plane resolution = 1  × 1 mm, slice thickness = 1 mm, imaging matrix = 256 × 256, repetition time (TR) = 2300 ms, echo time (TE) = 2.26 ms, inversion time (TI) = 900 ms, flip angle = 8°, field of view (FoV) = 256 mm, acquisition time = 5 min.

#### 2.3.2 Magnetic resonance spectroscopy

**FIGURE 1:**
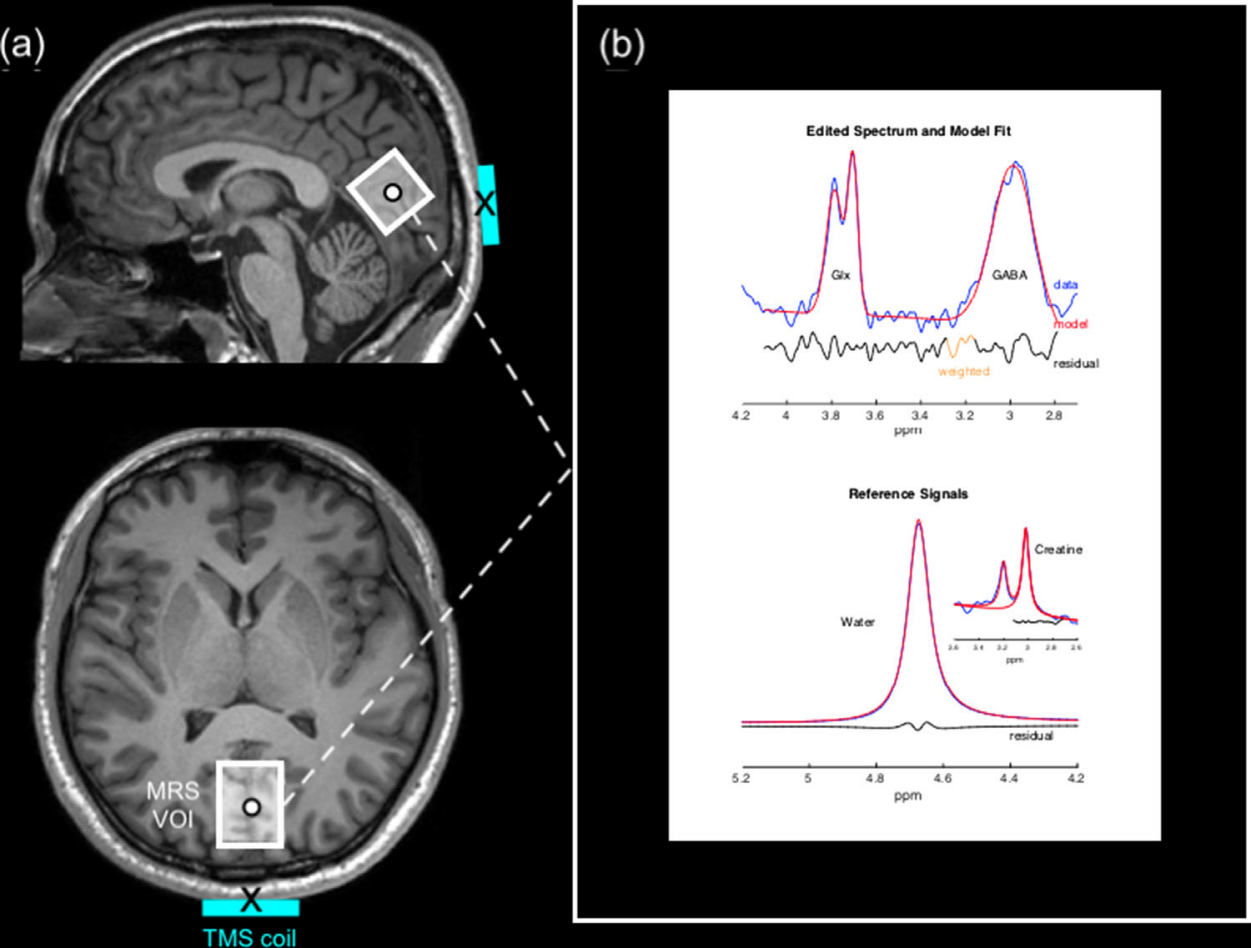
Positioning of the TMS coil and MRS VOI with example ^1^H MR spectra acquired from the visual cortex. (a) Example of standard voxel placement within the visual cortex on a T_1_‐weighted image for a single participant shown in the sagittal (top image) and transverse (bottom image) planes. Stimulation sites (black circle) were positioned at the center of the MRS VOI (white box) for each participant individually. The center of the TMS coil (black cross) was aligned to target the center of the MRS VOI. (b) An example of MEGA‐PRESS processing using Gannet. The blue lines indicate the difference‐edited spectrum, red line demonstrates a best fit Gaussian model, and the residual is shown in a black line. Upper plot shows the typical GABA peak that is observed at 3 ppm, and the Glx peak at 3.75 ppm. Lower plot shows Gannet modeling of the unsuppressed water signal and Cr signal against which GABA is quantified. ^1^H = proton; Cr = creatine; Glx = glutamate and glutamine composite; MEGA‐PRESS = Mescher‐Garwood Point Resolved Spectroscopy; MR = magnetic resonance; MRS = magnetic resonance spectroscopy; ppm = parts per million; TMS = transcranial magnetic stimulation; VOI = volume‐of‐interest

A single 25 mm^3^ cubic voxel was placed medially at the visual cortex (V1). The volume of interest (VOI) was placed as far back in the occipital pole's posterior region as possible and centered on the calcarine sulcus. The lower edge of the VOI followed the cortical surface, aligned alongside the cerebellar tentorium, and avoided non‐brain tissue (e.g., cerebrospinal fluid [CSF], sagittal sinus). The VOI position was verified in three planes (sagittal, coronal, and transverse) for accurate placement, and images in all planes were recorded and used as a reference for subsequent acquisitions for each participant. Proton (^1^H) MR spectra were obtained using the Mescher‐Garwood Point Resolved Spectroscopy (MEGA‐PRESS), a J‐ difference editing technique, through a C2P collaboration between Siemens and the University of Minnesota (CMRR), including a flip‐angle calibration procedure developed and recommended by the sequence developers. The following parameters were used for acquisition: TR = 3000 ms, TE = 68 ms, spectral width = 1500 Hz, sinc‐Gaussian editing pulses (nominal full‐width at half‐maximum [FWHM] bandwidth = 50.55 Hz) applied at 1.9 parts per million (ppm) (“edit‐on”) and 7.5 ppm (“edit‐off”), VAPOR water suppression (FWHM bandwidth = 60 Hz), averages = 32, repeated four times for a total of 128 “edit‐on” averages and 128 “edit‐off” averages, number of samples = 2048, acquisition time = 15 min. A separate unsuppressed water reference scan was also acquired to allow for a tissue concentration reference: averages = 10, total acquisition time = 52 min. Two automated shimming procedures were performed using Siemens B1 Shim mode and TrueForm. Figure 1a shows an example of the standard voxel placement in the occipital cortex. Figure 1b shows an example of the difference‐edited spectra for GABA+ and Glx peaks, as well as the creatine (Cr) signal in the “edit‐off” spectrum, and the water reference signal as plotted by Gannet software used to process and model the data (Edden et al., 2014).

### 2.4 TMS

A Magstim Rapid^2^ and Plus^1^ Stimulator 70‐mm diameter Double Air Film figure‐of‐eight coil and its sham coil counterpart (Magstim, Whiteland, Wales, UK) were used to deliver stimulation pulses or mimic stimulation pulses, respectively, to the defined target site.

#### 2.4.1 Phosphene threshold

Phosphene threshold (PT) is a method of measuring visual cortex excitability through the perception of phosphenes. A phosphene is a phenomenon of light that can be produced from direct stimulation of the occipital cortex in the absence of visual stimuli. PTs can be used to determine the individual intensity for TMS administration at the visual cortex in the same way that the motor threshold is used to determine TMS intensity when applied to the motor cortex. PT, therefore, provides an individual excitability threshold for TMS administration since thresholds vary greatly across individuals (Stewart et al., 2001).

In a dimly lit room, wearing a blindfold with eyes closed, participants were instructed to lean forward with their forehead resting on a table while placing no pressure on their eyes. Phosphenes are elicited when stimulation is applied from 1 to 5 cm above the inion and 0 to 3 cm laterally, depending on the hemisphere being tested (Elkin‐Frankston et al., 2010). We marked four locations to form a square area to be tested: at the inion, 2 cm above the inion, 2 cm to the left of the inion, and 2 cm above the 2 cm to the left of the inion marker. PTs were measured for each participant using single‐pulse stimulation with the coil center held tangential to the scalp and handle orientated at 90^0^ to the midline. The minimum output began at 50% intensity, and 10 pulses were administered to the marker 2 cm above the inion, with each pulse 6 s apart. Following a single TMS pulse, participants were instructed to respond “yes/no/maybe” corresponding to whether a phosphene was perceived that could vary in shape, color, motion, and size. At each location, the stimulator output was increased by 5% until phosphenes were evoked with the maximum output setting restricted to 90% intensity according to safety regulations (Wassermann, 1998). If no phosphenes were evoked after 10 pulses, the coil was moved to a new position in the marked region. The coil was placed in a new location until the individual responded “yes,” which was then designated as the hotspot. Subsequently, at the hotspot, the threshold was modified in 1% increments to refine the PT. A threshold was defined as the intensity at which 50% of pulses (5/10 pulses) resulted in a “yes” response. The blindfold was removed every 10−15 min, when necessary, for a minimum of 2 min, to prevent dark adaption (Boroojerdi et al., 2000). The range of reported PTs varied between 46% and 90% intensity (mean_PT_ = 65.31%).

#### 2.4.2 TBS

Participants underwent one of three TBS stimulation conditions performed at 80% PT: 1) cTBS, 2) iTBS, or 3) sham TBS. The cTBS protocol consisted of bursts containing three pulses at 50 Hz (20 ms between each stimulus), repeated at 5 Hz intervals (i.e., 200 ms inter‐stimulus interval [ISI]), applied continuously for 40 s, providing a total of 600 pulses. The iTBS protocol consisted of the same bursts containing three pulses at 50 Hz, repeated at 5 Hz intervals but applied in 2 s trains repeated every 10 s for a total of 190 s, also providing a total of 600 pulses (Huang et al., 2005). The sham TBS protocols were the same as the active conditions, except it was performed using the sham placebo coil. The sham coil is equipped with a shield that attenuates the magnetic field yet mimics auditory and stimulatory effects. Five individuals in the sham group experienced sham cTBS and four experienced sham iTBS.

TMS was delivered using Brainsight's neuronavigation system (Rogue Research, Montreal, QC, Canada). The target stimulation site corresponded to the center of the MRS VOI in V1 (Figure 1a). Participants’ anatomical MRI images were reconstructed and co‐registered to three‐dimensional cortical surfaces in Brainsight. The stimulation site was mapped on each participant's corresponding anatomical image in Brainsight by manually matching the anatomical landmarks to the MRS VOI images obtained at the baseline MRS acquisition. Reference points from the participant's head (tip of the nose, nasion, right and left tragus) were coregistered in Brainsight using the Polaris infrared image‐guided tracking system (Northern Digital Instruments, Kitchener, ON, Canada), which enables visualization and monitoring of stimulation in real‐time. Brainsight creates a co‐registration matrix using reference points from the MRI images and those marked on the participant's head that are tracked using Polaris to ensure accurately targeted stimulation throughout. Thus, the neuronavigation system precisely maps individually targeted stimulation sites and accounts for anatomical variability across participants. The coil was held parallel to the midline with the handle pointing downwards and the coil center tangential to the head to minimize coil to cortex distance as the participants sat upright with their chin resting on a chin rest.

### 2.5 Experimental design

**FIGURE 2:**
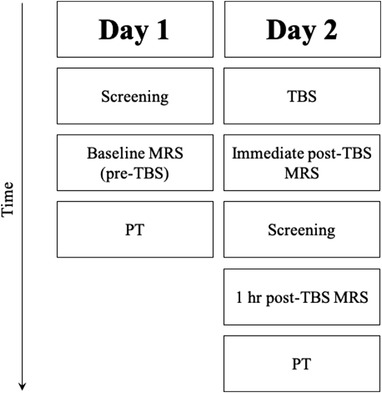
Diagram of the experimental procedure. Day 1) participants underwent visual and cognitive screening, followed by a baseline MRS scan, and lastly PT. Day 2) participants underwent one of three TBS conditions (continuous, intermittent, or sham), immediately followed by a MRS scan, repeated screening measures (including the addition of adverse effect reports), 1 h post‐TBS MRS, and finally repeat PT measures post‐TBS. MRS = magnetic resonance spectroscopy; PT = phosphene threshold; TBS = theta burst stimulation

The study was divided into two visits. Day 1) participants initially underwent pre‐TBS (baseline) screening consisting of eligibility questionnaires, vision assessments, and the MoCA at approximately 1 pm. MRS baseline measures were acquired upon eligibility at approximately 1:30 pm, followed by PT at approximately 2:30 pm. Day 2) participants received either cTBS, iTBS, sham iTBS (control) or sham cTBS (control) to V1. To minimize the potential diurnal variation of neuromodulators, including those involved in TMS mechanisms, the second visit was carried out approximately a week later as close to the baseline time of day as possible. At least 1 week was left between visits to limit any residual effects from the PT measurement interacting with TBS conditions while avoiding any long‐term fluctuations in metabolites. Metabolites are, however, reliably stable for at least several weeks (Henry et al., 2011; Near, Ho, et al., 2014). MRI acquisition began within 5 min of TBS cessation (immediate post‐TBS) and was repeated 1 h later (1 h post‐TBS) at visit 2. Vision assessments and the MoCA screening were repeated after the immediate post‐TBS scan and before the 1 h post‐TBS scan at the second visit. Participants were also asked to report any adverse effects of TBS. Finally, the PT was measured after the 1 h post‐TBS scan. Figure 2 shows an overview of the experimental procedure.

### 2.6 Magnetic resonance spectroscopy analyses

MRS data were processed using the MATLAB (The MathWorks Inc, Natick, MA; https://www.mathworks.com/products/matlab.html) based toolboxes Gannet (v3.0; http://www.gabamrs.com; Edden et al., 2014) and SPM12 (Statistical Parametric Mapping, Wellcome Centre for Human Neuroimaging, London, UK; http://www.fil.ion.ucl.ac.uk/spm).

All MRS data were pre‐processed with the default pipeline implemented in Gannet. The GannetLoad module processes time‐domain data including phased‐array channel combination, frequency‐and‐phase alignment of individual transients to mitigate effects of scanner frequency drift and subject motion, zero‐filling to 32,768 points, 3 Hz exponential line broadening, Fourier transformation, outlier rejection, and subtraction of the “on” and “off” spectra to generate the edited difference spectrum (Edden et al., 2014; Near, Edden, et al., 2014). Modeling of the different signals of interest was performed using the GannetFit module using non‐linear least‐squares optimization. The 3.02 ppm GABA+ signal in the difference spectrum was fit with a single Gaussian model, while the 3.75 ppm Glx doublet was fit with a double Gaussian model. The Cr reference signal in the “edit‐off” spectrum was obtained from a fit to the Cr and choline signals at 3.0 and 3.2 ppm, respectively, with a double Lorentzian model. The unsuppressed water signal was fit with a single mixed Gaussian‐Lorentzian model (Edden et al., 2014). The amplitude of the peak for each metabolite relates to the total number of molecules and represents the total concentration of that metabolite. GannetCoRegister invoked SPM12 to generate binary MRS voxel masks in the same image space as the T_1_‐weighted anatomical image. GannetSegment then calculated the relative tissue volume fractions from the voxel mask and segmentation results for grey matter (GM), white matter (WM), and CSF. Lastly, GannetQuantify used the amplitude parameters of the models and tissue volume fractions to calculate tissue‐corrected metabolite levels and derive quantitative measures of GABA+ and Glx concentrations in institutional units (i.u.). The final output value of GannetQuantify was used providing tissue‐corrected (relaxation‐ and alpha‐corrected, voxel‐average‐normalized) GABA+ and Glx concentration estimates relative to water. These corrections account for the effects of tissue composition, tissue water content, and water and metabolite relaxation, as well as the fact that GABA is present in higher concentrations in GM compared to WM at approximately a 2:1 ratio (Edden et al., 2014; Harris et al., 2015). We also include results for GABA+ and Glx concentrations as ratios relative to Cr, as is commonly reported in the literature, to allow the comparison of our data with a greater number of studies. Using Cr as an internal reference signal reduces the risk of error in the propagation of water‐based scaling, although care needs to be taken regardless of the choice of the reference signal as it is difficult to determine whether group differences arise from changes to the nominator or the denominator (Alger, 2010). For MEGA‐PRESS of GABA+, water‐referenced quantification including tissue‐correction shows similar levels of performance to Cr referencing, and the reliability of the two referencing strategies is comparable (Mikkelsen et al., 2017, 2019). For data quality control, datasets with a fit error over 10% (defined as the standard deviation of the fit residual divided by the model amplitude) or visible subtraction artifacts in the difference spectrum were removed (Mikkelsen et al., 2019). This resulted in the removal of all time points for one participant as mentioned in Section 2.1.

### 2.7 Statistical analyses

Statistical analyses were conducted in R statistical software (v1.1.456; R Foundation for Statistical Computing, Vienna, Austria; www.R‐project.org). Data were found to violate assumptions of parametric testing. Multilevel mixed modeling was used since it not only accounts for the non‐parametric nature of data, but also for repeated measures, is highly flexible in dealing with varying intervals between measurements, and can deal with unequal sample sizes appropriately. Akaike's information criterion was used to measure the goodness of a fit of an estimated model, and the appropriate covariance structure with the lowest reported criterion was used for statistical analysis. Tissue fractions within the VOI across visits were analyzed as no significant changes would indicate consistent VOI positioning across visits and groups. Multilevel modeling was performed for each tissue fraction (GM, WM, CSF) separately with random effect for participant, and fixed effect for TBS condition (cTBS, iTBS, sham) and visit (pre‐TBS, immediate post‐TBS, 1 h post‐TBS). The effect of TBS conditions on metabolites was also performed using multilevel modeling for GABA+ and Glx concentrations separately, with random effect for participant, and fixed effect for TBS condition (cTBS, iTBS, sham) and visit (pre‐TBS, immediate post‐TBS, 1 h post‐TBS). Post hoc analyses were performed using Yuen's *t*‐tests (YW) for non‐normally distributed data with 10% trimmed means. Effect sizes for Yuen (ES_YW_) were also calculated using 10% trimmed means. The relationship between PT (visual cortical excitability), GABA+, and Glx concentrations was analyzed using the nonparametric correlation Kendall's tau (*τ*). For all statistical analyses, the significance level was set at *p *< .05, and corrected for multiple comparisons using the false discovery rate procedure where relevant.

## 3 RESULTS

### 3.1 Tissue fractions

For GM, there was no significant interaction between TBS condition and visit, *F*(4, 52) = 1.191, *p* = 0.326; no significant main effect of TBS condition *F*(2, 26) = 0.528, *p* = 0.596; and no significant main effect of visit *F*(2, 52) = 0.3, *p* = 0.745.

For WM, there was no significant interaction between TBS condition and visit, *F*(4, 52) = 2.237, *p* = 0.078; no significant main effect of TBS condition *F*(2, 26) = 0.654, *p* = 0.528; and a significant main effect of visit *F*(2, 52) = 4.1, *p* = 0.022. However, post hoc analysis showed no significant differences in WM between pre‐TBS and immediate post‐TBS visits, YW(24) = −0.518, *p* = 0.609, ES*_YW_* = 0.02; no significant difference between pre‐TBS and 1 h post‐TBS, YW(24) = −1.358, *p* = 0.419, ES_YW_ = 0.08; and no significant difference between immediate post‐TBS and 1 h post‐TBS, YW(24) = −1.108, *p* = 0.419, ES_YW_ = 0.06.

For CSF, there was no significant interaction between TBS condition and visit, *F*(4, 52) = 1.105, *p* = 0.364; no significant main effect of TBS condition *F*(2, 26) = 0.237, *p* = 0.79; and no significant main effect of visit *F*(2, 52) = 1.437, *p* = 0.247.

### 3.2 GABA+ concentrations

**FIGURE 3:**
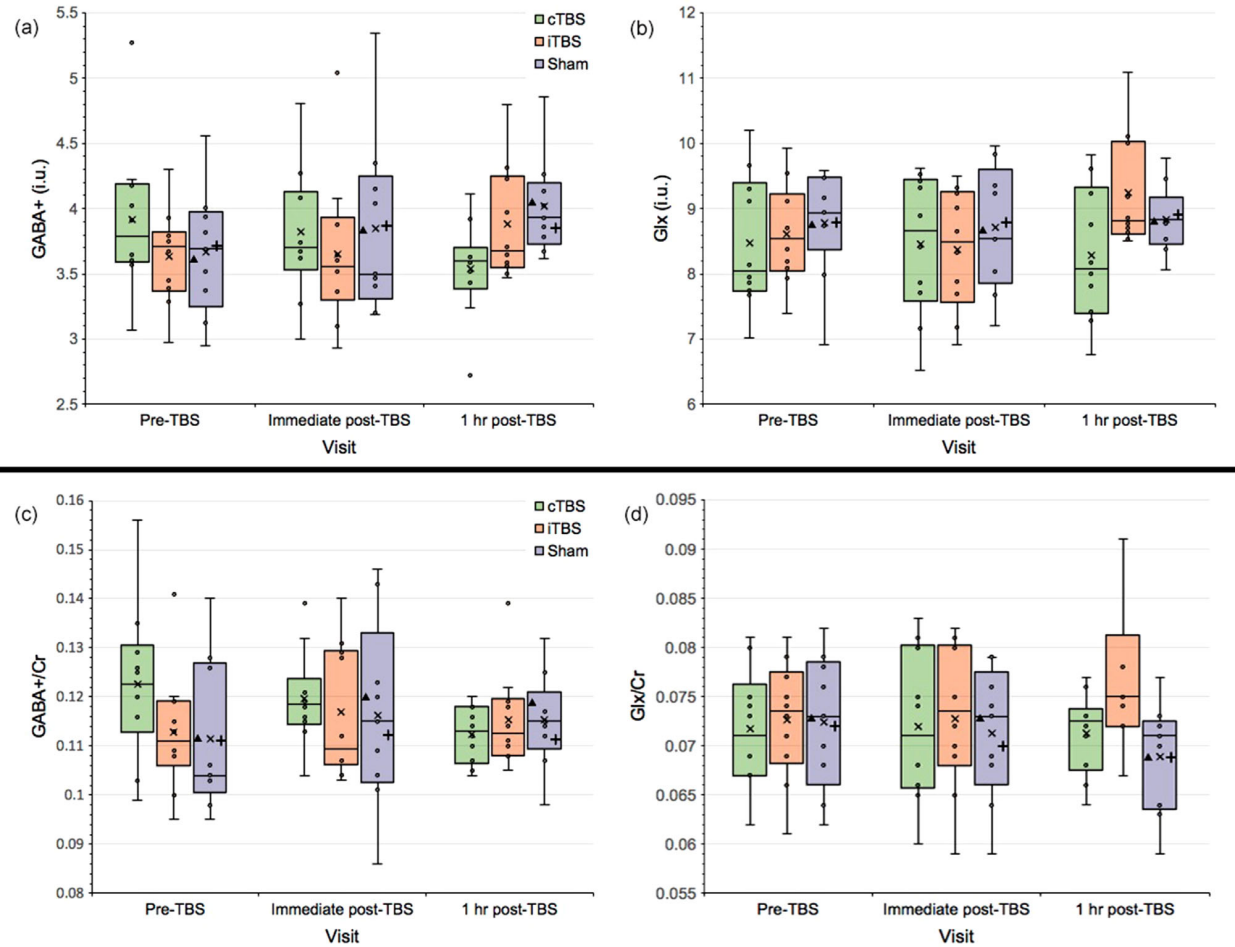
Changes in GABA+ and Glx concentrations at the visual cortex following cTBS and iTBS sessions across all visits expressed as (a,b) tissue‐corrected values (i.u.), and (c,d) normalized concentrations using integral ratios relative to Cr. Box plots show the exclusive interquartile range. Extreme points represent outliers. Symbols: *x* represents the group mean; ▴represents the mean for the sham cTBS group; + represents the mean for the sham iTBS group. GABA+ = GABA and macromolecules composite; Glx = glutamate and glutamine composite; Cr = creatine; i.u. = institutional units; cTBS/iTBS = continuous/intermittent theta burst stimulation

For tissue‐corrected GABA+ (i.u.), there was no significant interaction between TBS condition and visit, *F*(4, 52) = 1.899, *p* = 0.125; no significant main effect of TBS condition *F*(2, 26) = 0.96, *p* = 0.396; and no significant main effect of visit *F*(2, 52) = 1.775, *p* = 0.18. Figure 3a shows GABA+ tissue‐corrected concentrations across all time points.

For GABA+/Cr, there was similarly no significant interaction between TBS condition and visit, *F*(4, 52) = 1.083, *p* = 0.374; no significant main effect of TBS condition *F*(2, 26) = 2.047, *p* = 0.149; and no significant main effect of visit *F*(2, 52) = 2.049, *p* = 0.139. Figure 3c shows GABA+/Cr concentrations across all time points.

### 3.3 Glx concentration

For tissue‐corrected Glx (i.u.), there was no significant interaction between TBS condition and visit, *F*(4, 52) = 1.363, *p* = 0.26; no significant main effect of TBS condition *F*(2, 26) = 0.277, *p* = 0.761; and no significant main effect of visit *F*(2, 52) = 0.152, *p* = 0.859. Figure 3b shows Glx tissue‐corrected concentrations across all time points.

For Glx/Cr, there was similarly no significant interaction between TBS condition and visit, *F*(4, 52) = 1.087, *p* = 0.373; no significant main effect of TBS condition *F*(2, 26) = 0.0004, *p* = 1.0; and no significant main effect of visit *F*(2, 52) = 0.0002, *p* = 1.0. Figure 3d shows Glx/Cr concentrations across all time points.

### 3.4 Correlation between GABA+, Glx, and phosphene threshold

**FIGURE 4:**
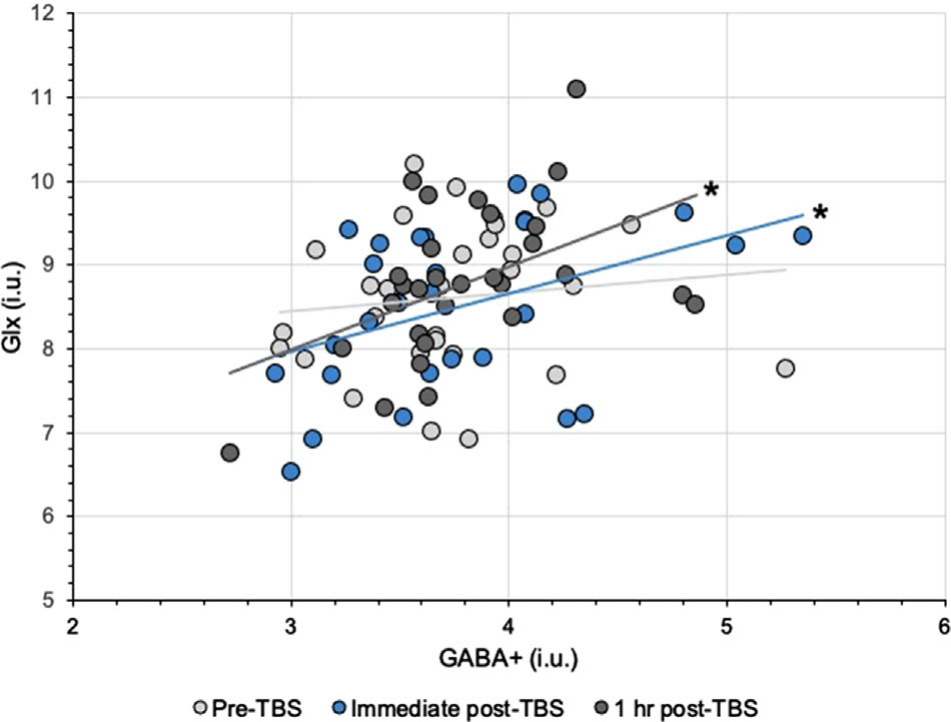
Changes in correlation between tissue‐corrected GABA+ and Glx concentrations (i.u.) at the visual cortex following TBS conditions across all visits. * *p* < .05. GABA+ = GABA and macromolecules composite; Glx = glutamate and glutamine composite; i.u. = institutional units; TBS = theta burst stimulation

Since there were no significant changes following either of the TBS conditions, the following measures were collapsed across TBS conditions/groups. There were no significant correlations between pre‐TBS GABA+ and Glx concentrations (i.u.), *τ* = −0.149, *p* = 0.507; pre‐TBS PT and GABA+ concentrations (i.u.), *τ* = 0.082, *p* = 0.535; and pre‐TBS PT and Glx concentrations (i.u.), *τ* = −0.127, *p* = 0.507. Similarly, there were no significant correlations between 1 h post‐TBS PT and GABA+ concentrations (i.u.), *τ* = −0.055, *p* = 0.955; and 1 h post‐TBS PT and Glx concentrations (i.u.), *τ* = −0.008, *p* = 0.955. However, there were significant correlations between immediate post‐TBS GABA+ and Glx concentrations (i.u.), *τ* = 0.315, *p* = 0.017; and 1 h post‐TBS GABA+ and Glx concentrations (i.u.), *τ* = 0.319, *p* = 0.047. Figure 4 shows GABA+ and Glx correlations following TBS across all time points.

### 3.5 Adverse effects

Following TBS, one participant in the cTBS condition and one participant in the sham condition reported a minor headache, and one participant in the sham group reported craniofacial discomfort near the left eye. These effects are consistent with common and temporary reports following TMS (Oberman et al., 2011; Rossi et al., 2009).

## 4 DISCUSSION

This study is the first to provide data on the immediate and short‐term effects of cTBS and iTBS to the visual cortex on V1 GABA and glutamate concentrations. We found that a single session of cTBS or iTBS had no significant effects on MRS measures of GABA+ and Glx levels and visual cortical excitability at a cohort level. However, TBS did alter the relationship between GABA+ and Glx for up to 1 h following stimulation. These preliminary findings suggest that unlike TBS to the motor or frontal cortices, TBS to the visual cortex can be used in investigative or clinical settings without significant implications or alterations to these neurotransmitter levels at the stimulation site.

These effects following cTBS to the visual cortex are consistent with our previous findings where a single session of conventional “inhibitory” 1 Hz rTMS to the visual cortex also did not affect GABA+ and Glx concentrations (Rafique & Steeves, 2020). There are no reports in the literature investigating the effects of conventional “excitatory” high‐frequency rTMS to the visual cortex on GABA+ and Glx to allow a comparison with our iTBS findings. Although TBS is frequently reported to produce similar effects to conventional rTMS, effects on epidural volleys in the corticospinal pathway demonstrate that the after‐effects are not homogenous across the TMS protocols. Each TMS paradigm modulates specific neural elements in different layers of the cortex. The cTBS protocol suppresses the amplitude of the I1 wave, suggesting that cTBS has its major effect on the synapse between the inputs responsible for the I1 wave and the pyramidal tract neurons, whereas 1 Hz rTMS produces a selective suppression of late I waves with no change in the I1 wave. In contrast to cTBS, the iTBS protocol produces a selective enhancement of late I waves with no change in the amplitude of the I1 wave (for a review, see Di Lazzaro & Rothwell, 2014).

Although we found no significant effects on absolute levels of GABA+ and Glx, our results do not simply imply that TBS had no effects on LTD or LTP or associated metabolites at the visual cortex. Pre‐TBS, there was no significant relationship between GABA+ and Glx measures. There was, however, a significant relationship between these metabolites following TBS that lasted up to 1 h post‐TBS. This finding may suggest that TBS caused subtle changes in the relationship between the metabolites, one that was not sufficiently large to cause discernible changes between the conditions. Stagg et al. (2011) found a significant positive correlation between MRS‐measures of GABA and glutamate at the stimulation site following TMS to the motor cortex, where glutamate measures were an indicator of global motor excitability. The authors suggest that the tight biochemical relationship between the neurotransmitters may be driven by glutamate since glutamate is a precursor to GABA. While MRS‐measured glutamate is considered to reflect synaptic glutamatergic activity (Stagg & Nitsche, 2011), MRS measures of GABA represent the total sum of GABA_A_ and GABA_B_ receptor activity (inhibitory and excitatory activity, respectively) (Luo et al., 2011; Rae, 2014). GABA_B_ receptor mechanisms are facilitated to an extent by glutamatergic activity (Chalifoux & Carter, 2011; Prout & Eisen, 1994). The effects of TBS are also in part mediated by glutamatergic mechanisms (Huang et al., 2011). Since it is still unclear how TBS protocols precisely interact with LTP and LTD mechanisms, if TBS acts on glutamatergic mechanisms, then it may affect the overall homeostatic balance between GABA and glutamate concentrations rather than simply impacting one metabolite over the other. Donahue et al. (2010) have used a number of haemodynamic approaches to determine the relationship between GABA and cerebral blood measures. In the presence of higher GABA concentrations, they suggest that the greater associated vascular and metabolic response occurs presumably to promote an increase in excitatory activity required to overcome the inhibition that may result from higher GABA availability. The lack of causal effects in our study may also be masked by other factors. We observed high interindividual variability across participants in the present study. Offline NIBS protocols tend to demonstrate larger intra‐ and interindividual variability in their aftereffects, which are dependent on numerous factors. The current brain state at the time of stimulation, history of synaptic activity, structural asymmetry, neurochemistry, the specific interneuron networks recruited, hormonal levels, circadian rhythms, sex, age, genetics (e.g., polymorphisms) are but some of the factors influencing interindividual variability (Hamada et al., 2013; Miniussi et al., 2013; Polania et al., 2018; Ridding & Ziemann, 2010; Silvanto et al., 2007). Although we attempted to constrain these variables as much as possible in our participant recruitment, there are factors that we could not control for that may have led to null findings, for example, structural differences and genetics. Intraindividual variability is, however, considerably consistent over weeks (Hinder et al., 2014; Vernet et al., 2014). The null finding in the present study may, therefore, be partially owed to the fact that NIBS triggers a complex chain of effects confounded by several variables, some of which are not directly observable (Bergmann & Hartwigsen, 2021).

Our findings stand in contrast to Allen and colleagues (2014) who found that cTBS significantly increased GABA+ at V1 following stimulation. One aspect of the contrasting finding could be owed to differences in MRI scanner and acquisition parameters. The most notable disparities include differences in stimulation coil, inclusion criteria, threshold determination, and active versus passive viewing during MRS. We used a figure‐of‐eight coil to deliver stimulation, which has been shown to produce more focal effects than the circular coil used by Allen et al. (2014); however, some circular coils may be capable of producing deeper effects depending on the geometry and design (Deng et al., 2013). We employed much stricter exclusion criteria to control for confounding variables associated with TMS and metabolites to minimize interindividual variability mentioned above that substantially impacts the response to TMS. Full details on our exclusion/inclusion criteria are provided in Section 2.1. Allen et al. (2014) used the MT rather than the PT to determine stimulation intensity. We used the PT as it offers a more accurate and relevant measure of visual cortical excitability, and quantifies suprathreshold stimulation of target neurons at the visual cortex. The MT is shown to not accurately reflect visual cortical excitability (Boroojerdi et al., 2002; Gerwig et al., 2003; Stewart et al., 2001). Further, MTs are markedly lower than PTs, which would result in lower stimulation intensity in the study by Allen and colleagues. When the intensity of TMS is lowered, a reversal of TMS effects has been demonstrated (Abrahamyan et al., 2011). It is recommended that at least allowances in intensity should be made to account for the cortical distance from the motor cortex and associated changes in neural tissue when using the MT for non‐motor regions to minimize the risk of substantial under‐ or overstimulation (Stokes et al., 2007, 2005). Lastly, Allen et al. (2014) employed an active viewing task where participants watched a film during MRS acquisition, whereas our acquisition was performed at rest with the eyes closed in a dark room. Task viewing stimulates cortical activity (Vanderwal et al., 2017) and activates large‐scale brain networks (van der Meer et al., 2020) that can impact metabolite levels as has been shown previously for MRS measures of GABA and Glx (Duncan et al., 2014; Kurcyus et al., 2018). Thus, task viewing in itself may have impacted metabolite levels and/or interacted with stimulation effects.

We also found that TBS had no significant impact on visual cortical excitability using 80% PT. Two studies using a circular coil have found that cTBS increased PTs when applied at 80% PT (Allen et al., 2014; Franca et al., 2006), and iTBS had no effect (Franca et al., 2006). Conversely, Brückner and Kammer (2016) have found that a figure‐of‐eight coil significantly decreased PTs following cTBS applied at 80% PT, whereas a circular coil had no significant effect. At least with corticospinal excitability, thresholds are higher with a circular rather than figure‐of‐eight coil; however, reliability is better for the figure‐of‐eight than circular coil (Fleming et al., 2012). No significant change in PT is observed following cTBS or iTBS at 100% PT using a figure‐of‐eight coil, but a change in PT is seen following cTBS that is related to whether a visual acuity task is presented (Brückner & Kammer, 2015). A significant change in PT occurs when presented with high visual demands following cTBS, but no change in PT is observed in a low visual demand condition (i.e., rest), consistent with our protocol and our data. Stochastic resonance, a phenomenon that exists in systems with measurement thresholds, may account for these observed differences (Schwarzkopf et al., 2011). The phenomenon suggests that information is enhanced by the injection of low levels of noise that in turn lower the response threshold, whereas higher noise levels disrupt performance. Brückner and Kammer (2016) propose that cTBS to the visual cortex at 80% of individual PT using a figure‐of‐eight‐coil may add low levels of noise to the visual system, thereby lowering the PT compared to the circular coil that has more diffuse effects and can depolarize a greater number of neurons. Using a higher intensity of 100% PT may exceed the amount of noise that would improve signal detection in the stochastic resonance framework. Reducing the stimulation intensity to 80% of PT may correspondingly reduce the volume of depolarized neurons differently and the resultant network modulation would be different (Brückner & Kammer, 2016). Subthreshold TMS is associated with opposing effects compared with suprathreshold TMS (Nahas et al., 2001; Nakamura et al., 1997). Consequently, the choice of threshold, as well as the coil, likely affects any impact on metabolite levels. However, the inhibitory effect following cTBS at 80% PT is only apparent in participants with higher PTs, with the slope of baseline PT predicting the direction of modulation irrespective of coil type (Brückner & Kammer, 2016). For participants with lower baseline PTs, there may be low levels of noise to the system, thus, increasing excitability in an already excitable system and lowering the threshold for both circular and figure‐of‐eight coils. In an individual with a higher PT and reduced visual cortical excitability (Áfra et al., 1998; Aurora & Welch, 1998; Terhune et al., 2015), there may be larger background neuronal activity. Such elevated baseline noise in participants with higher PTs could be increased further by TBS as per the stochastic resonance phenomenon and would lead to increased PTs. Particularly with the round coil, the induced noise would be expected to be greater as well as stimulating a greater number of neurons. Additionally, a visual acuity task (i.e., high visual demand) following TBS modifies the state‐dependent neuronal effects (Pasley et al., 2009; Perini et al., 2012), including raising noise levels that may have led to increased PTs (Brückner & Kammer, 2016). Optimal levels of noise are necessary to push weak subthreshold signals over the threshold, thereby improving information transfer.

Continued investigation into the neurophysiological effects of NIBS will allow us to refine the poorly generalized assumption that stimulation interaction with underlying brain activity, structure, and its ability to target specific neuronal pathways is homogenous across the brain. Cortical excitability and neural noise depend not only on the stimulation parameters but also the morphophysiological properties of the stimulated area, spontaneous and task‐induced brain states, and intrinsic connectivity patterns (Fertonani & Miniussi, 2017; Nettekoven et al., 2015; Silvanto & Muggleton, 2008). Stimulating cortical and subcortical surfaces with TMS induces changes in a mixture of neuronal populations that utilize distinct neurotransmitters and perform specific actions/functions. These physiological, functional, and anatomical properties as well as connectivity profiles greatly influence the diverse responses to stimulation depending on the site of stimulation. The concept of “excitatory” effects of iTBS and “inhibitory” effects of cTBS are shown to be highly variable even at the motor cortex depending on differences in the interneuronal cortical networks that are preferentially recruited by the TMS pulse (Hamada et al., 2013) and the subregion stimulated (Martin et al., 2006). LTP and LTD in the visual cortex vary depending on the layer in the visual cortex that is stimulated since each layer depends on specific receptor and neurotransmitter activation (Daw et al., 2004). Identical stimulation protocols induce a differential cascade of effects since not all brain regions respond equally (Castrillon et al., 2020; Funke & Benali, 2010). It has been suggested that the effects of NIBS are determined by the extent of functional integration of a target region rather than the frequency range of the stimulation protocol (Castrillon et al., 2020). Numerous studies choose only to employ cTBS and not iTBS based on the findings of Huang et al. (2005) that demonstrate the effect of iTBS was not as strong nor as long‐lasting as cTBS. Franca et al. (2006) also report that iTBS does not have effects at the visual cortex; however, we have described earlier why findings differ between TBS studies to the visual cortex and, therefore, why conclusions regarding iTBS null effects are not so straightforward. iTBS protocols may simply need modification to produce maximal effects. For example, iTBS effects are strongly related to baseline network connectivity (Nettekoven et al., 2014). Priming and conditioning with opposing cTBS and iTBS protocols have been shown to magnify the conditioned aftereffects (Murakami et al., 2012). Quite simply, we do not know enough about TMS protocols or how to maximize their associated effects. Prior to developing a clinical intervention protocol or implying causality in investigative research using TMS, mechanistic knowledge about TMS processes and its contribution to changes in brain regions and networks following stimulation needs to be better quantified in healthy populations. Initial work in healthy participants can lead to translational validation studies for therapeutic use in visual disorders such as amblyopia, visual hallucinations, and other visual impairments. Monitoring of symptoms using neurophysiological data longer term is needed to substantiate mechanisms and optimize stimulation parameters. Direct validation can then begin in target patient populations by refining stimulation parameters depending on the cortical/subcortical target site and the patient population in question to begin to ameliorate pathophysiological mechanisms. Beyond the approved use of TMS for a limited number of conditions, for example, medication‐resistant depression, and more recently obsessive‐compulsive disorder, and smoking cessation, there is a lack of approved therapeutic NIBS protocols due to the shortage of studies investigating effects at non‐motor or frontal cortices.

### 4.1 Limitations and future directions

The immediate and shorter‐lasting effects of TBS may be lost during the acquisition of anatomical images and time taken for VOI positioning that cannot be overcome (∼10 min in total and was consistent across participants). Additionally, the limited spectral resolution at clinical field strengths (∼3T) hampers the reliable separation of glutamate resonances from glutamine, and the composite measure of Glx may dilute the effects of glutamate. However, Glx is considered primarily driven by the glutamate signal as glutamate is found at much greater concentrations than glutamine in the brain (Stagg, 2014). While the edited GABA signal is also contaminated with co‐detected spectra, variations in macromolecules between individuals are estimated to be small (Hofmann et al., 2001; Kreis et al., 2005). As such, it is unlikely that macromolecule contamination affects interpretability. Importantly, MRS GABA estimates are reliable across weeks at the occipital cortex (Near, Ho, et al., 2014), and show low interindividual variability, but are not observed to correlate across cortical regions (Greenhouse et al., 2016). Glutamate concentrations are considered stable for at least 1 month (Henry et al., 2011). Overall, GABA and glutamate concentrations show excellent reproducibility in the visual cortex using the MEGA‐PRESS sequence (Evans et al., 2010; Henry et al., 2011; O'Gorman et al., 2011).

Since TMS focality is not confined to the stimulation site but also regions nearby as well as inducing direct and indirect distal effects on neurophysiological and cognitive behavior, it is essential to measure stimulation effects in distal brain regions. Given the multifactorial nature of variables contributing to TMS effects, this will help to establish how TMS affects metabolites across the brain, provide more insight into effects on biochemical pathways, and enable fine‐tuning of parameters appropriately. Vidal‐Piñeiro et al. (2015) have previously found no change in GABA at the stimulation site but found significant changes distally. Due to the single voxel limit of MRS, this requires further separate sessions of the highly controlled experimental design with identical stimulation protocols.

We used 80% threshold intensity in line with the original TBS protocol (Huang et al., 2005) and due to uncertainty over safety at higher intensities especially at the visual cortex since PTs are generally higher than MTs (Boroojerdi et al., 2002; Gerwig et al., 2003; Stewart et al., 2001). In pathophysiology, greater unpredictability to NIBS is expected (Maeda et al., 2000; Wassermann, 2002). Studies have safely employed TBS up to 120% MT to the frontal cortex in disease states without reducing tolerability (Bakker et al., 2015; Fregni et al., 2006; McClintock et al., 2017). In addition, intensities of 100% and 120% PT have been used safely at the visual cortex (Brückner & Kammer, 2015, 2016). Since the response to TMS varies depending on whether subthreshold, threshold, or suprathreshold intensities are employed, it is important to establish whether cTBS and iTBS have differing effects on V1 GABA+ and Glx at 100% and even 120% PT.

The present study and our previous rTMS study (Rafique et al., 2020) are not directly comparable due to differences in the total number of pulses (600 versus 1200 pulses, respectively). Offline protocols of rTMS tend to be longer in order to produce longer‐lasting changes (Thut & Pascual‐Leone, 2010). The general analogy that TBS produces similar effects to rTMS does not account for the discrepancy in that the number of pulses is usually unequal. Previous studies have demonstrated that doubling the duration of stimulation can produce a contrasting effect (Gamboa et al., 2011, 2010; Goldsworthy et al., 2015). Future work is required to compare equivalent protocols in terms of pulses between conventional rTMS and TBS at the visual cortex since analogous effects may not be seen when the number of pulses is equal. The effects of accelerated/within‐session TBS also need to be studied at the visual cortex. Accelerated rTMS produces accumulative, stable, and longer‐lasting effects than a single session (Rafique & Steeves, 2020) and single daily sessions over consecutive days/weeks (Goldsworthy et al., 2014; Holtzheimer et al., 2010). Accelerated TBS is considered safe and well tolerated in clinical populations (Desmyter et al., 2014; Duprat et al., 2016), but investigations at the visual cortex and in visual‐related disorders are crucially lacking.

## 5 CONCLUSION

We demonstrate that cTBS and iTBS protocols at the visual cortex have different effects than those seen at the motor and frontal cortices. With the protocol employed in this study, neither paradigm caused significant shifts in visual cortex GABA+ or Glx. TBS did, however, change the relationship between visual cortex GABA+ and Glx up to 1 h post‐TBS suggesting that effects may be subtly sufficient to alter the relationship between metabolites but not induce significant changes to the metabolite concentrations. Modified protocols of TBS may be needed at the visual cortex to produce substantial changes in LTD and LTP mechanisms, particularly if the tool is to hold value for translation to therapeutic use in patient populations.

### PEER REVIEW

The peer review history for this article is available at https://publons.com/publon/10.1002/brb3.2478
